# A Clinical Conundrum of Unilateral Ovarian Agenesis With Associated Ipsilateral Absence of the Fallopian Tube

**DOI:** 10.7759/cureus.99016

**Published:** 2025-12-11

**Authors:** Sumaira Rafique, Suganya Sukumaran

**Affiliations:** 1 Obstetrics and Gynaecology, George Eliot Hospital, Nuneaton, GBR

**Keywords:** absence of fallopian tube, haematosalpinx, heterotopic pregnancy, suboptimal decline in bhcg, unilateral ovarian agenesis

## Abstract

Unilateral ovarian agenesis (UOA) is a rare condition that is often associated with the ipsilateral absence of the fallopian tube.

We report the case of a 31-year-old woman, G3P1+1, at approximately 5 weeks’ gestation, who initially presented with left iliac fossa pain, raising concern for a possible ectopic pregnancy versus miscarriage. The differential diagnosis was later expanded to include heterotopic pregnancy with left haematosalpinx, based on early imaging findings and a suboptimal decline in serial serum beta-human chorionic gonadotropin (β-hCG) levels. As a result, an urgent laparoscopic evaluation with evacuation of retained products of conception (ERPOC) was performed, which confirmed the absence of the left ovary and fallopian tube, along with an intrauterine anembryonic pregnancy.

This case highlights the need for a high index of suspicion when imaging findings and β-hCG trends do not correlate, and underscores the importance of a systematic laparoscopic examination for definitive diagnosis.

## Introduction

Unilateral ovarian agenesis (UOA) is a rare condition that is often associated with the ipsilateral absence of the fallopian tube, with an estimated rate of 1 in 11,240 [1]. Reported causes include congenital absence of the paramesonephric ducts, unilateral torsion, or vascular insult and compromise at any stage of life [1,2]. However, UOA is not consistently linked to miscarriage or infertility, and a normal pregnancy is equally possible [3,4]. As a result, this condition may go undetected or may be identified incidentally on imaging, during abdominal surgery, or at laparoscopic evaluation [5], sometimes prompted by inconclusive imaging studies.

## Case presentation

Our patient is a 31-year-old White British woman, G3P1+1 (with one previous child aged two years), who presented to the early pregnancy assessment unit (EPAU) at around 5 weeks’ gestation with mild-to-moderate left iliac fossa pain for four days. She reported no constitutional symptoms such as fever, weight loss, night sweats, dizziness, or shoulder tip pain. Other important negatives included no per-vaginal (PV) spotting or discharge, no urinary symptoms, and no change in bowel habits. She had not experienced any recent trauma or participated in contact sports.

She was otherwise well and a non-smoker. Her past medical and surgical history was unremarkable, aside from one previous miscarriage of unclear cause. She had no history of sexually transmitted infections (STIs) and was in a stable long-term relationship with a single partner. There was no personal or family history of genetic conditions or tuberculosis.

On examination, the patient was apyrexial with stable vital signs. Her abdomen was soft, with tenderness in the left iliac fossa only on deep palpation. The remainder of the general and systemic examination was unremarkable.

**Figure 1:**
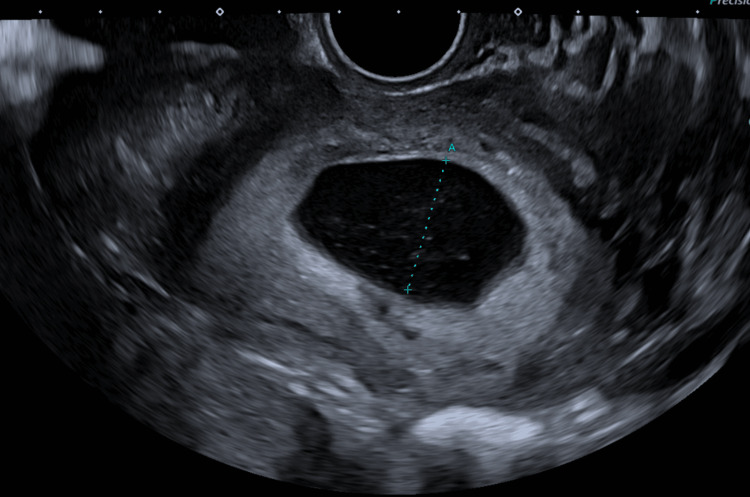
Transvaginal ultrasound showing an anembryonic pregnancy. This image shows an irregular sac within the uterine cavity with no visible embryo or foetal pole.

**Figure 2:**
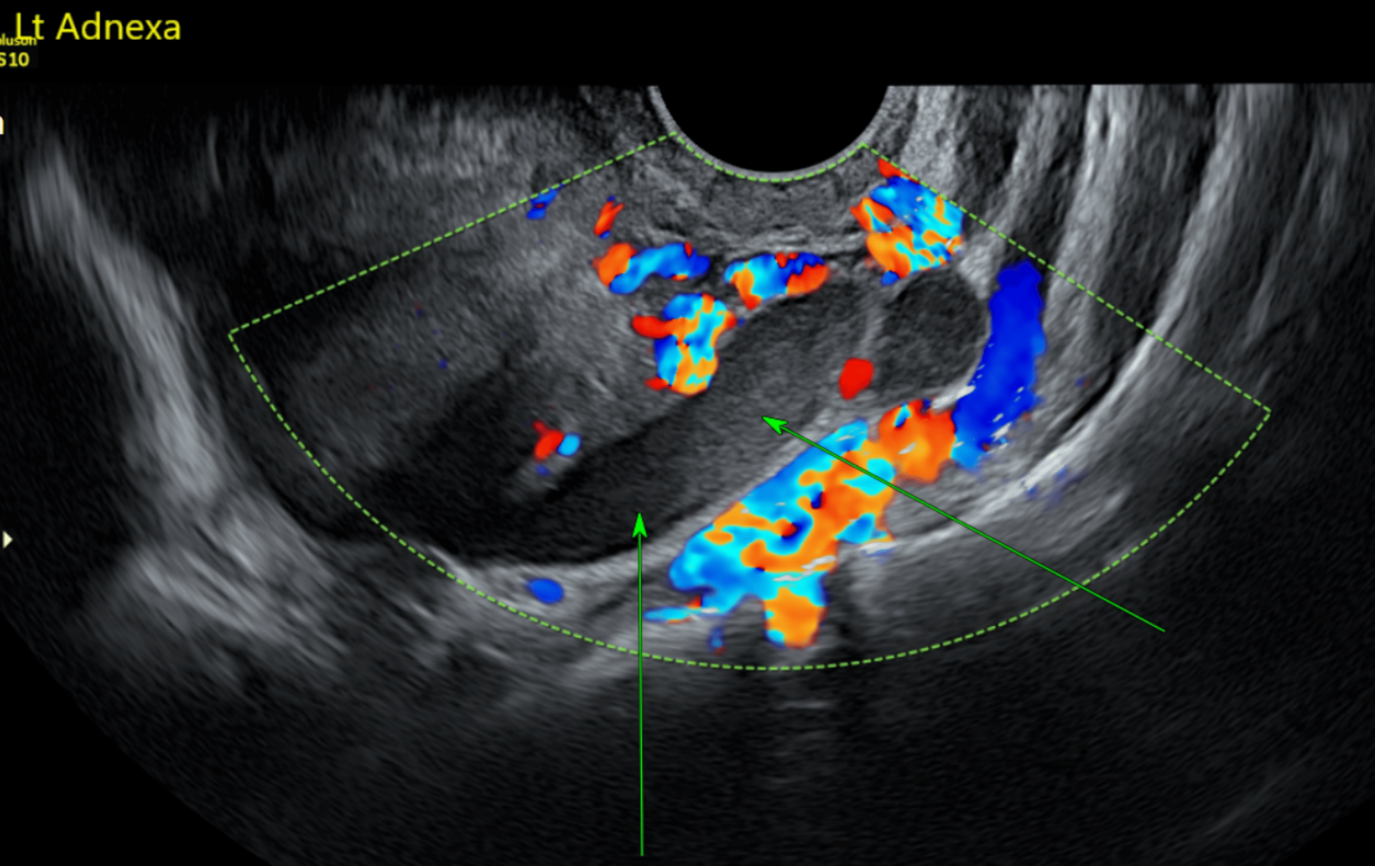
Transvaginal ultrasound showing a tubular structure consistent with a haematosalpinx.

Serial serum β-hCG showed a suboptimal decrease from the initial level of 127,670 IU/L. Other blood test results were unremarkable. Ultrasound demonstrated an intrauterine anembryonic pregnancy along with a haematosalpinx in the left adnexa (Figures 1, 2).

**Figure 3:**
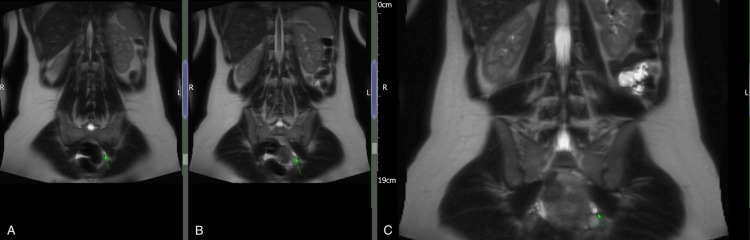
(A-C) Coronal T2-weighted MRI sequences showing a tubular structure in the left adnexa, raising suspicion for a haematosalpinx.

**Figure 4:**
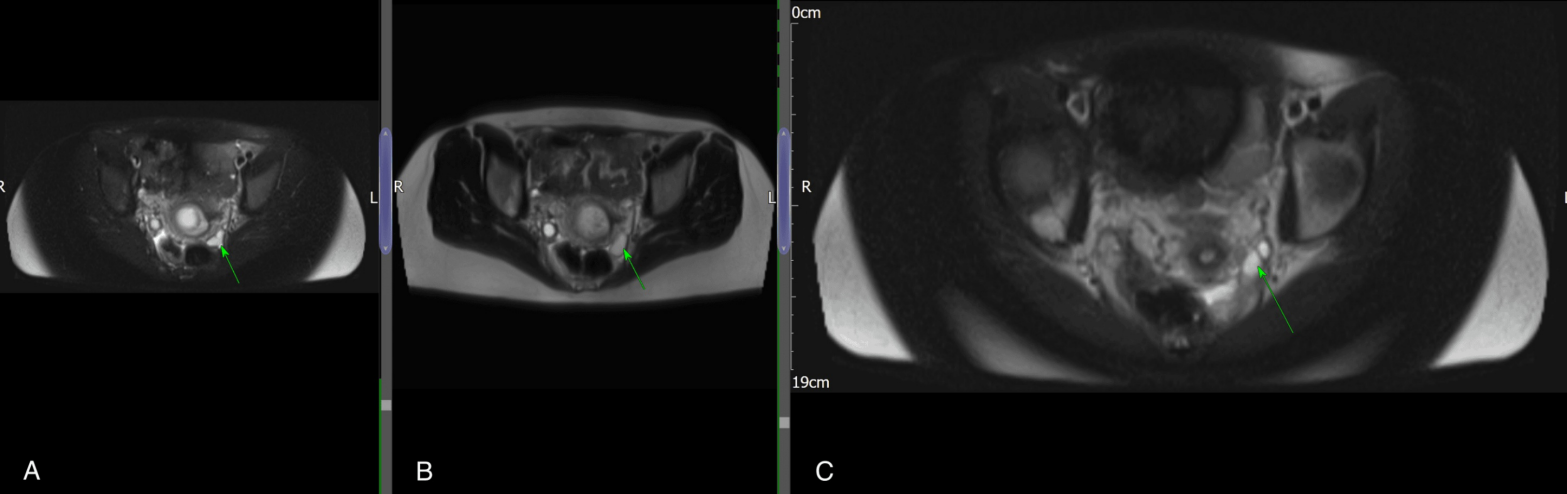
(A-C) Axial T2-weighted MRI sequences showing the sequential appearance of a tubular structure in the left adnexa, raising suspicion for a haematosalpinx.

Following the ultrasound, MRI showed a tubular structure in the left adnexa with intermediate signal on T2-weighted images (Figures 3, 4) and high signal on T1-weighted images, suggesting a possible haematosalpinx.

**Figure 5:**
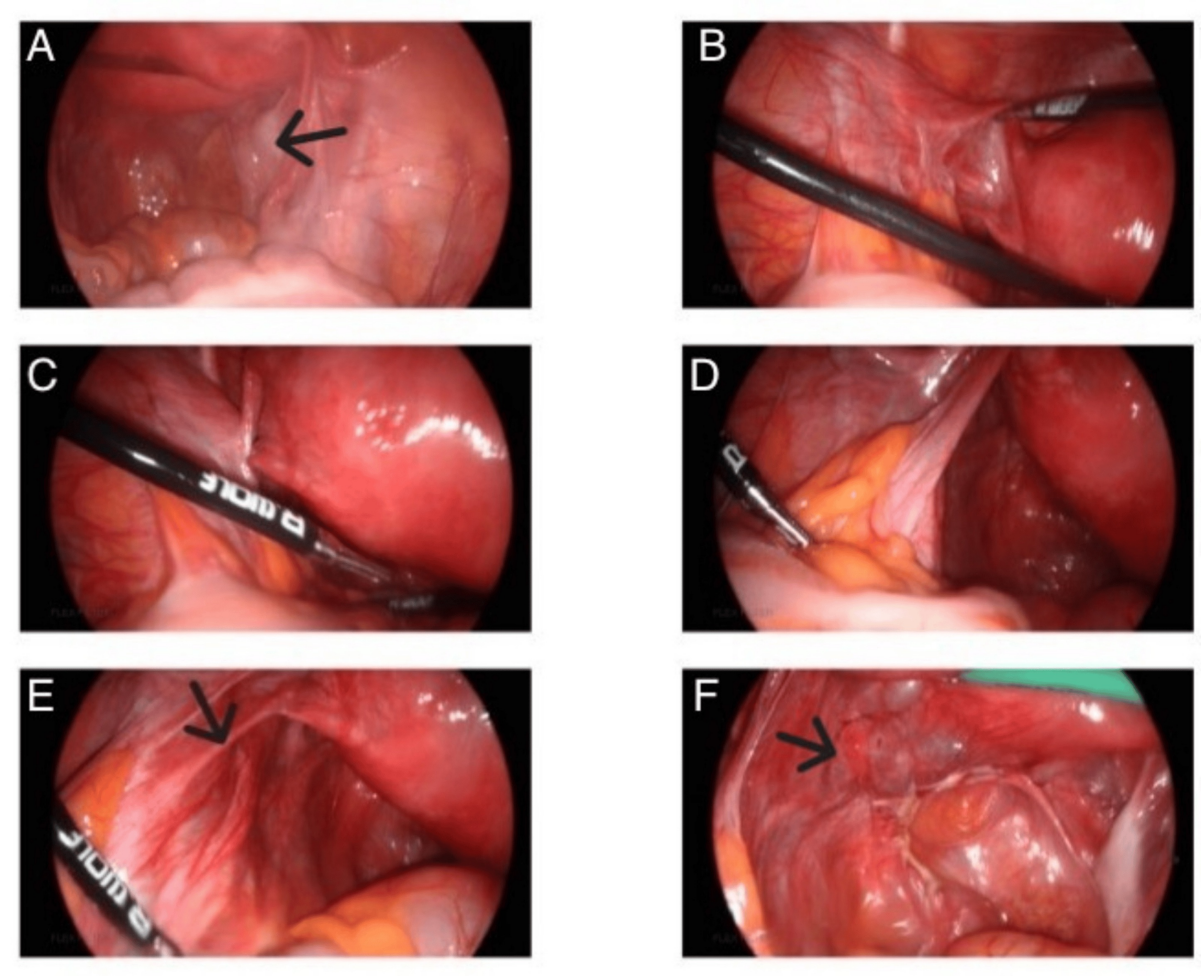
(A-E) Laparoscopic images showing the absence of the left ovary and fallopian tube. Black arrows: (A) right ovary, (E) left round ligament, (F) pelvic veins.

This prompted consideration of laparoscopy as part of the diagnostic and therapeutic approach. Laparoscopy confirmed the incidental absence of the left ovary and fallopian tube (Figure 5), and the anembryonic pregnancy was managed simultaneously through evacuation of the products of conception.

The patient had an uneventful postoperative recovery and was discharged the following day. Serum β-hCG levels repeated after 48 hours showed an appropriate decline, and subsequent serial measurements continued to fall until becoming negative. Histopathology of the evacuated tissue confirmed products of conception consistent with an anembryonic pregnancy.

At three months of follow-up, the patient achieved a new spontaneous conception and went on to deliver a healthy baby at 12 months, marking a reassuring and positive outcome to her clinical course.

## Discussion

UOA is a rare condition, with most studies describing torsion or vascular insult as the most common aetiology, followed by indeterminate causes. Embryological factors, such as true congenital agenesis, are reported less frequently. Differentiating congenital agenesis from vascular autoamputation remains challenging [1,2,5].

UOA may present with abdominopelvic pain or infertility and is often associated with additional anatomical abnormalities. Ipsilateral absence of the fallopian tube is reported in approximately 81% of cases, and concomitant uterine (26%) and renal (20%) anomalies also occur [3,4]. The rarity of unilateral ovarian and fallopian tube absence may introduce diagnostic expectation bias, while imaging accuracy depends on the skill of the operator and interpreter [3]. Therefore, a higher index of suspicion is warranted when either ovary is not clearly visualised across multiple imaging studies. During laparoscopy, it is also critical to rule out an ectopic (ectopic-positioned) ovary along its developmental descent path [3].

UOA may also be identified incidentally, such as during surgery for other indications (e.g., caesarean section), with or without associated renal anomalies. Additionally, renal anomalies may occur independently in the foetus without maternal manifestations [5]. Fertility in women with unilateral ovarian absence is generally considered minimally affected or unaffected, with infertility rates reported around 19%-21% [3,6]. Although smaller case series have suggested higher infertility rates [6], larger reviews support that normal conception and pregnancy are common [3,4]. This aligns with our patient’s history of a previous uncomplicated pregnancy, indicating that unilateral ovarian absence does not necessarily impair reproductive potential.

Our case was unique and challenging because the left ovary was not visualised, yet the presence of an intrauterine anembryonic pregnancy along with a left adnexal tubular structure suggested a haematosalpinx on both ultrasound and MRI (Figures 1-4). This raised immediate concern for heterotopic pregnancy, an urgent and potentially catastrophic diagnosis that must not be missed when imaging appears suspicious [7]. The suboptimal decline in β-hCG further heightened concern, prompting urgent laparoscopy. Intra-operatively, a complete absence of the left ovary and ipsilateral fallopian tube was confirmed, and the adnexal structure previously thought to represent a haematosalpinx was identified as dilated pelvic veins (Figure 5).

The gold standard for diagnosis is diagnostic laparoscopy, and a “three-cycle inspection approach” is strongly recommended. This begins with upper abdominal inspection in a clockwise fashion before placing the patient in the Trendelenburg position and inserting accessory trocars. This is followed by inspection of the pelvic brim to identify key anatomical landmarks, major vessels, and the ureter. Finally, a detailed examination of the pelvic organs and internal genitalia is performed, often with the help of a uterine manipulator. Although generally safe, laparoscopy carries surgical risks and may be unnecessary in the absence of additional clinical indications [8].

## Conclusions

UOA with associated absence of the fallopian tube is a rare condition. It can present a significant diagnostic challenge, and a high index of suspicion is needed to differentiate it from a heterotopic pregnancy, particularly when normal gynaecological anatomy is not confirmed on routine imaging and when there is a suboptimal decline in β-hCG. In such cases, early consideration of a thorough laparoscopic assessment and ERPOC is warranted.

During laparoscopy, a systematic inspection of the abdomen and pelvis is essential. Following an organised approach is a key learning point to ensure that unilateral absence of the ovary and fallopian tube is not overlooked, especially when imaging is equivocal or fails to clearly identify one or both adnexal structures.
